# Effect of Combined Prenatal and Adult Benzophenone-3 Dermal Exposure on Factors Regulating Neurodegenerative Processes, Blood Hormone Levels, and Hematological Parameters in Female Rats

**DOI:** 10.1007/s12640-020-00163-7

**Published:** 2020-01-23

**Authors:** Alicja Skórkowska, Alicja Maciejska, Bartosz Pomierny, Weronika Krzyżanowska, Beata Starek-Świechowicz, Beata Bystrowska, Żaneta Broniowska, Grzegorz Kazek, Bogusława Budziszewska

**Affiliations:** 1 Department of Biochemical Toxicology, Medical College, Jagiellonian University, Medyczna 9, 30-688 Krakow, Poland; 2 Department of Toxicology, Chair of Toxicology, Medical College, Jagiellonian University, Medyczna 9, 30-688 Krakow, Poland; 3 Department of Pharmacodynamics, Medical College, Jagiellonian University, Medyczna 9, 30-688 Krakow, Poland

**Keywords:** Benzophenone-3, Frontal cortex, Hippocampus, Neurotoxicity, Glutamate, Thyroid hormones, Hematological parameters

## Abstract

Benzophenone-3 (BP-3), the most widely used UV chemical filter, is absorbed well through the skin and gastrointestinal tract and can affect some body functions, including the survival of nerve cells. Previously, we showed that BP-3 evoked a neurotoxic effect in male rats, but since the effects of this compound are known to depend on gender, the aim of the present study was to show the concentration and potential neurotoxic action of this compound in the female rat brain. BP-3 was administered dermally to female rats during pregnancy, and then in the 7th and 8th weeks of age to their female offspring. The effect of BP-3 exposure on short-term and spatial memory, its concentrations in blood, the liver, the frontal cortex, and the hippocampus, and the effect on selected markers of brain damage were determined. Also, the impact of BP-3 on sex and thyroid hormone levels in blood and hematological parameters was examined. It has been found that this compound was present in blood and brain structures in females at a lower concentration than in males. BP-3 in both examined brain structures increased extracellular glutamate concentration and enhanced lipid peroxidation, but did not induce the apoptotic process. The tested compound also evoked hyperthyroidism and decreased the blood progesterone level and the number of erythrocytes. The presented data indicated that, after the same exposure to BP-3, this compound was at a lower concentration in the female brain than in that of the males. Although BP-3 did not induce apoptosis in the hippocampus and frontal cortex, the increased extracellular glutamate concentration and lipid peroxidation, as well as impaired spatial memory, suggested that this compound also had adverse effects in the female brain yet was weaker than in males. In contrast to the weaker effects of the BP-3 on females than the brain of males, this compound affected the endocrine system and evoked a disturbance in hematological parameters more strongly than in male rats.

## Introduction

2-hydroxy-4-methoxybenzophenone (benzophenone-3; BP-3) is the most widely used UV-absorbing compound. This compound is included not only in sunscreen formulas but also in most other cosmetics and is used as a stabilizer in plastic food packaging materials. BP-3 absorbs well through the skin from preparations containing it, and also from the gastrointestinal tract from contaminated food or water (Janjua et al. 2004). This compound is considered to be a pollutant of the water reservoir environment, accumulates in aquatic organisms, and, at a high concentration, is found in fish fat (Fent et al. 2010). In people, BP-3 is absorbed well through the skin and is detected in serum in concentrations ten times higher than other chemical filters, plus the presence of its metabolites has been demonstrated in about 98% of urine samples (Calafat et al. 2008; Janjua et al. 2008; Tarazona et al. 2013).

Benzophenones belong to the group of endocrine-disrupting chemicals (EDCs) and affect the function of sex and thyroid hormones. So far, the effects of BP-3 on estrogen receptors (ER) have been studied best, and both in vivo and in vitro studies indicate the agonistic effect of this compound on the ER. It was also found that BP-3 exhibited an antagonistic effect on the human androgen receptor (AR) and progesterone receptors (PR) and may contribute to fertility reduction in both women and men, and in the development of hormone-dependent tumors (Ma et al. 2003; Schlumpf et al. 2001, 2004; Schreurs et al. 2005).

In contrast to the relatively well-characterized BP-3 action on the function of the gonads, little data relates to the action of this compound on brain cells. When considering the increasing exposure of people to this compound, the possibility of its interfering with the central effects of steroid and thyroid hormones, and data indicating that compounds from the EDCs group may damage neurons, BP-3 may prove to be an important factor involved in the induction or intensification of cell damage observed in neurodegenerative diseases. So far, the neuron-damaging effects of BP-3 were demonstrated in vitro (Broniowska et al. 2016; Wnuk et al. 2018a), in vivo in the brains of mice embryos after prenatal exposure (Wnuk et al. 2018b, 2019), and in our studies in adult male rats after combined prenatal and adult dermal exposure (Krzyżanowska et al. 2018; Pomierny et al. 2019).In our previous studies, dermal administration of BP-3 in both the prenatal period, when the brain cells are the most sensitive to neurotoxic factors, and for 2 weeks to adult male rats, we showed pro-apoptotic changes in the frontal cortex and CA1 region of the hippocampus, and most likely this action was related to elevated levels of extracellular glutamate (Krzyżanowska et al. 2018; Pomierny et al. 2019). Because it was found that after dermal exposure to BP-3, with higher concentrations of this compound in human beings found in the blood of males than females, it seems that the neuronal damaging effects of BP-3 may also be dependent on gender (Janjua et al. 2008). Therefore, the aim of the present study was to show whether, after the same exposure to BP-3 as previously used in the case of males, there are differences in female rats in the concentration of this compound in the blood, in its level in the brain, and in the damage to brain cells. In order to determine the mechanism of BP-3 action in the central nervous system of female rats, its effect on factors involved in neuronal damage or protection processes, such as (1) increase in extracellular glutamate concentrations and expression of its transporters, (2) activation of microglial cells, (3) lipid peroxidation intensification, (4) expression of sex hormone receptors mediating the neuroprotective hormones’ effect, and (5) the level of the arylhydrocarbon receptor (AhR) involved in the neurodegenerative activity of many EDCs was investigated in the frontal cortex and hippocampus. These brain structures were selected because they are most vulnerable to damage and have key involvement in the cognitive processes. Moreover, to assess memory deficits, short-term and spatial memory was determined in a new object and new location recognition test. In the current study, BP-3 was administered dermally due to this being the main route of exposure in humans. This compound was applied in the prenatal period and during the first 2 weeks of adulthood because, firstly, we wanted to compare its effects in females with previously tested effects in males and, secondly, this pattern of administration is the best way to observe the brain cell damage. A lot of current research indicates that exposure to xenobiotics in the prenatal period sensitizes brain cells and leads to stronger damaging effects of this compound acting later in life (Li et al. 2014; Lien et al. 2015; Modgil et al. 2014).

Endocrine-disrupting chemicals, including UV filters from the benzophenone group, in addition to the effects on the sex hormones, most often also disturb the function of the thyroid gland (Broniowska et al. 2018; Hofmann et al. 2009). In the case of sex hormones, EDCs usually affect the function of their receptors without changing the level of the hormones themselves, while in the case of thyroid hormones, these compounds rather interfere with their synthesis. To determine the effect of BP-3 on gonadal hormone, blood concentrations of 17β-estradiol, progesterone, and testosterone were determined. Since prolactin by inhibition of gonadotropins is a common cause of low sex hormone levels, its concentration was also determined. To determine the activity of the hypothalamic-pituitary-thyroid (HPT) axis, the concentrations of free fractions of triiodothyronine (fT3) and thyroxin (fT4), and the thyroid-stimulating hormone (TSH), were determined. Free hormone fractions were assayed, because their blood levels better reflect the thyroid function than the total of T3 and T4.

To date, there is no evidence on the impact of BP-3 on the number and morphology of blood cells. However, one benzophenone derivative from the fruit of *Garcinia indica* (garcinol) has been shown to affect the membrane of human erythrocytes and causes cell shrinkage (Fazio et al. 2015). Previously, we found that another benzophenone derivative, benzophenone-2, neither changed the leukocyte, erythrocyte, or platelet count nor affected the morphology and hemoglobin content in erythrocytes, although this compound was administered to adult, male rats and additionally exhibits less cytotoxic activity than BP-3 (Broniowska et al. 2018). Therefore, the next aim of this study was to evaluate the effects of BP-3 on the hematological parameters of peripheral blood in female rats. We determined the red blood cell (RBC) count, mean corpuscular volume (MCV), hematocrit (HCT), hemoglobin concentration (HGB), mean cell hemoglobin concentration (MCHC), leukocyte count (WBC), and platelet count (PLT).

## Materials and Methods

### Animals and Treatment

The experiments were performed on Sprague Dawley rats from the animal housing facility of the Jagiellonian University Medical College in Cracow. The animals were kept under a natural day-night cycle at 22 ± 2 °C with food and water available at libitum.

To determine estrous cycle phases, vaginal smears were taken from the females on a daily basis. On the proestrus day, the females were placed with males for 12 h, and the vaginal smears were subsequently examined for the presence of sperm.

BP-3 (purity-98%) obtained from Merck (Darmstadt, Germany) was formulated daily in Essex cream (Schering-Plough, Brussels, Belgium) at a final concentration of 10%, and administered from the first to the last day of pregnancy (approx. 22–23 days). Prior to treatment, the hair on the back of the neck to halfway towards the tail region was shaved and the animals were reshaved during treatment when the hair began to reappear. The examined group was treated with cream containing BP-3, at a dose of 100 mg/kg twice daily (at 8:00 and 17:00), whereas control female rats were treated with Essex cream without BP-3. We used a dose of 100 mg/kg BP-3, because after this dose, BP-3 blood levels in rats are comparable to those seen in humans using preparations containing this compound. It was shown that in humans after dermal application of a formulation containing 5% BP-3, the maximum concentration of these compound in plasma was 200–300 μg/l, and after 24 h about 80–200 μg/l (Tarazona et al., 2013). Similarly, Janjua et al. (2008) found that after a single application of BP-3, its concentration in the serum in men was about 250 ng/ml. We observed comparable BP-3 blood levels in the male rat, i.e. 216 ng/ml after the 100-mg/kg dose (Pomierny et al., 2019). After birth, the offspring were kept with the dam without any treatment. Twenty-one days after birth, male and female offspring were weaned and housed in groups of five per cage (with the males and females being separated) under standard conditions, without any treatment until the 43rd day. From 43 to 56 days of age, the female rats whose mothers received BP-3 were administered dermally with this compound, whereas cream without BP-3 was given to the control animals. The animals were observed daily for any abnormalities and their weights were recorded weekly. Two hours after the last morning BP-3 (or vehicle) application, the animals were subjected to the novel object recognition test or the novel location of the object recognition test. All tests were performed on 2-month-old females weighing 227.4 ± 15,5 g (control group) and 230.3 ± 12.8 g (BP-3 group). On the next day (24 h after the last BP-3 administration), the animals were euthanized via rapid decapitation. A small volume of the trunk blood was collected either in heparinized tubes for the hematological determination or the remaining volume in tubes containing EDTA, and these blood samples were centrifuged at 800×g, at 4 °C for 15 min and then plasma was stored at − 80 °C until used for biochemical assays. The brains and livers were rapidly removed, and the brain structures (hippocampus and frontal cortex) were dissected on ice-cold glass plates, frozen on dry ice, and stored at − 80 °C.

### Novel Object and Novel Location Recognition Tests

To determine the impact of BP-3 on cognitive function (short-term and spatial memory), female rats from control and BP-3-treated groups were subjected to a novel object or novel location recognition test (Jabłoński et al. 2013). During habituation, the animals were allowed to explore an empty container and 24 h later they were exposed for 5 min to the same area containing two identical objects placed at an equal distance. Next, the animals were subdivided into two groups and 1 h after the pretest, rats from the first group were allowed to explore the same container for 5 min in the presence of the familiar object and a novel object, consistent in height and volume but different in shape and appearance. Animals from the second group were placed in the same container equipped with two identical familiar objects, but one of them was located in another place. The tests were recorded. After the tests were conducted, the films were manually checked to calculate the time that the animals spent exploring both objects. The results of the tests were presented as the preference index, i.e., the ratio of the time the animal spent exploring a novel object (for the novel object test) or exploring the object located in another place (for the novel location test) to the time spent exploring of both object.

### LC/MS Analysis of BP-3

Tissues were homogenized in deionized water. Samples of homogenate or plasma mixed with internal standard were subjected to extraction by adding heptane and dichloromethane (1:1; *v*/v) and shaking for 10 min on an oscillating shaker. After centrifugation (10 min at 4000×g), the organic layer was obtained and evaporated under nitrogen stream at 37 °C.

Next, the chromatographic separation with mass spectrometric analysis was performed using Agilent 1100 liquid chromatograph (Agilent, Waldbronn, Germany) coupled to a mass spectrometer API 2000 (triple quadrupole) (Applied Biosystems MDS Sciex, Concord, Ontario, Canada) equipped with an electrospray ionization (ESI) interface. Chromatography equipment included a degasser, a binary pump, an autosampler and a column (Thermo Scientific BDS Hypersil C18; 100 × 3 mm I.D., 3 μm particle size) thermostated at 30 °C with its precolumn (100 × 3 mm I.D., 3 μm particle size) was used to separate samples—the volume of the sample injection was 40 μl with the flow rate of 0.4 ml/min. The mobile phase compositions were 0.025% glacial acetic acid in water (A) and methanol (B) with gradient: 0–1.5 min, isocratic gradient 40.0% (B); 1.5–2,5 min, linear gradient 40.0–95.0% (B); 2.5–6,5 min, isocratic gradient 95.0% (B); 6.5–8,0 min, linear gradient 95.0–40.0% (B); 8.0–10.0 min, and isocratic gradient 40.0% (B).

High-purity nitrogen (99.9%) from Peak NM20ZA as a curtain and collision gas and electrospray ionization in positive mode were used in the mass spectrometry analysis. The following parameters of ion source were applied: ion spray voltage (IS) 5000 V; nebulizer gas (gas 1) 20 psi; turbo gas (gas 2): 10 psi; temperature of the heated nebulizer (TEM) 250 °C; curtain gas (CUR) 20 psi, and the next ion path parameters for BP-3, BP-1, and BP-d10 were declustering potential (DP) 8 V; focusing potential (FP) 10 V; collision cell entrance potential (CEP) 13 V; and collision cell exit potential (CXP) 18 V. The following pairs of ions were monitored (values of m/z in brackets): BP-3 (229.0/151.1), BP-1 (215.0/107.0), and BP-d10 (193.0/110.0). For data analysis, Analyst software 1.6 (Perlan Technologies) was used. BP-3 and BP-1 concentrations were computed using calibration curves, constructed by linear regression analysis of the peak area versus concentrations.

### Total Antioxidant Capacity

The total antioxidant capacity was determined using a modification of the ORAC method previously described by Sofic et al. (2002) and Prior and Cao (1999). Within this assay, the loss of fluorescein (as a probe) fluorescence is measured over time due to peroxyl-radical formation by the breakdown of 2,2′-azobis-2-methyl-propanimidamide dihydrochloride (AAPH). The tissue antioxidants inhibit this reaction. 6-Hydroxsy-2,5,7,8-tetramethylchroman-2-carboxylic acid (TROLOX) was used as a standard in the concentration range from 2.5 to 200 μM. The results were calculated according to Prior and Cao (1999), using the differences in areas under the fluorescein decay curves between the blank and a sample, and extrapolated to the TROLOX standard curve prepared in the same manner. Data are expressed as a percentage of the control group.

### Lipid Peroxidation (MDA) Level

Fluorimetric assays for lipid peroxidation were performed using the Lipid Peroxidation (MDA) Colorimetric/Fluorometric Assays Kit (BioVision, USA). The assay is based on the reaction of the main product of lipid peroxidation, malondialdehyde (MDA) with thiobarbituric acid (TBA), at 95 °C for 60 min. The product of this reaction is MDA-TBA adduct, which can be quantified fluorometrically (Ex/Em = 532/553). The fluorescence was measured by a fluorescence plate reader (Fluoroskan Ascent FL, Thermo Labsystems). Lipid peroxidation in the samples was calculated from the standard curve and gathered as nanomoles of MDA per milligram of protein. The results were recalculated and expressed as a percentage of the control group.

### Microdialysis of Frontal Cortex and Hippocampus

After 2.5% isoflurane anesthesia guide cannulas were stereotaxically implanted in the animals’ frontal cortex (anteroposterior (AP) −0.48 mm; mediolateral (ML) +2.0 mm; dorsoventral (DV) −1,2 mm—according to the atlas of Paxinos and Watson (2007), and the hippocampus (AP −4.36 mm; ML +1.8 mm; DV −2.5 mm)) using dental acrylic cement and cranial screws. Next, the cannulas were supported with obturators and 24 h later microdialysis was performed. Afterwards, the obturators were removed and, before collecting the first baseline sample, microdialysis probes (MAB 4, membrane with a molecular weight of 6 kDa cutoff, 2 mm length and 0.24 mm outer diameter, AgnTho’s AB, Sweden) were perfused with artificial cerebrospinal fluid (aCSF components [mM]: NaCl 147, KCl 4.0, MgCl_2_ 1.0, CaCl_2_ 2.2, pH 7.4) for 2 h at a constant flow rate of 2 μl per minute. Then, microdialysis probes were inserted into the guide cannulas of the frontal cortex and hippocampus before microdialysis samples were collected every 30 min for 3 h and immediately frozen and kept at a temperature of − 80 °C until further chromatographic analysis.

### LC-MS Analysis of Glu

The chromatographic separation with mass spectrometric analysis was performed using the Agilent HPLC 1100 series system (Agilent, Waldbronn, Germany). Chromatographic separation parameters were based on Jastrzębska et al. (2015). Chromatography equipment included a degasser, a binary pump, an autosampler, and a thermostated column compartment. LiChrospher 60 RP-select B column (125 mm × 4.6 mm ID, 5 μm particle size) and a suitable guard column (4 mm × 4 mm, 5 μm particle size) (Merck, Germany) were used to separate aCSF samples. Following this, pairs of ions were monitored (values of m/z in brackets): Glu (148.0/84.1), Glu-d_5_ (153.22/89.1). For data analysis, Analyst software 1.4 (AB SCIEX, USA) was used. Glu concentrations in the noted brain structures were computed using calibration curves, constructed by linear regression analysis of the peak area versus concentrations. The presented data are expressed as the means of six samples collected every 30 min during 3 h of microdialysis for each animal.

### RT-PCR Method

The female rats were decapitated 24 h after the last BP-3 administration; their brains were immediately removed, and the hippocampus and frontal cortex were isolated and immersed in RNAlater solution (Ambion, USA). According to the manufacturer’s protocol, the total RNA was extracted using TRI Reagent (Zymo Research, USA) and purified with Direct-zol RNA Miniprep Kit (Zymo Research, USA). Reverse transcription reactions and real-time PCR were conducted using a Transcriptor First Strand cDNA Synthesis kit and Fast Start Universal Probe Master (ROX) (Roche, Germany). According to the manufacturer’s protocol and using the PrimeQ real-time PCR system expression of the GLT-1, xc^−^, ERα, ERβ, GPR-30, AhR, C1qb, Cd40, Aif, and the reference gene GAPDH was analyzed. The fold change in expression was determined using the ΔΔc(t) method of relative quantification.

### Western Blot Analysis

To obtain the membrane, nucleus, cytosol, and cytoskeleton fractions, the frontal cortex and hippocampus were subjected to subcellular fractionation using the Subcellular Protein Fractionation Kit (Roche, Germany). The protein concentrations were determined by the BCA Protein Assay Kit (Thermo, USA), and the individual cell fractions after dilution to the same protein concentration were mixed with a loading buffer (1:1) and heated for 5 min at 95 °C. Afterwards, samples containing 30–50 μg of the total protein in 15 μl were loaded on the stain-free gradient 4–15% SDS polyacrylamide gels (Bio-Rad, USA) and the electrophoresis was performed for 1 H at 150 V. The total amount of protein was evaluated in gels using the G:BOX Imaging System (Syngene, USA). Then, the proteins were transferred to PVDF membranes using a Trans-Blot Turbo Transfer System (Bio-Rad, USA). After several washes and blocking non-specific binding sites in 5% BSA, the membranes were incubated overnight with the following primary antibodies (in 1% BSA): anti-GLT-1 (EAAT2), anti-xc^−^, anti-ERα, anti-ERβ, anti-GPR30, anti-PR, anti-AhR, anti-Caspase 3 active, anti-Caspase 8, anti- Caspase 9, anti-Bax, anti-Bcl-2, anti-Iba1, anti-Caspase-1, anti-RIP, anti-glutathione (GPx) (Abcam (UK) or Santa Cruz Biotechnology (USA)). The membranes were then washed several times and incubated for 1 H at room temperature with proper secondary antibodies (in 1% BSA): goat anti-mouse-HRP, 1:7500, sc2005, Santa Cruz Biotechnology (USA); and goat anti-rabbit-HRP, 1:10000, ab6721, Abcam (UK) (Table 1). After washing, the signals were developed using the ECL method (Western Bright Quantum, Advansta, USA) and acquired by the G:BOX Imaging System (Syngene, USA). Making use of Gene Tools software (Syngene, USA), the protein expression was analyzed, taking into account the total amount of protein loaded into each well.

**Table 1:** List of antibodies with their concentrations and vendors used in this study

Target molecule	Dilution	Catalog number	Manufacturer
Western blot—primary antibodies			
GLT-1 (EAAT2)	1:3000	ab41621	Abcam (UK)
xc^−^	1:2000	ab37185	Abcam (UK)
ERα	1:400	sc-787	Santa Cruz Biotechnology (USA)
ERβ	1:1000	ab3576	Abcam (UK)
GPR30	1:2000	ab39742	Abcam (UK)
AhR	1:200	sc-133,088	Santa Cruz Biotechnology (USA)
Caspase-3 active	1:1000	ab2302	Abcam (UK)
Caspase-8 active	1:400	sc-56,070	Santa Cruz Biotechnology (USA)
Caspase-9	1:400	sc-17,784	Santa Cruz Biotechnology (USA)
Bax	1:2000	ab32503	Abcam (UK)
Bcl-2	1:400	sc-7382	Santa Cruz Biotechnology (USA)
Caspase-1	1:500	ab179515	Abcam (UK)
RIP1	1:500	ab72139	Abcam (UK)
GPx	1:200	sc-22,145	Santa Cruz Biotechnology (USA)
Western blot—secondary antibodies			
Goat anti-mouse-HRP	1:7500	sc-2005	Santa Cruz Biotechnology (USA)
Goat anti-rabbit-HRP	1:10000	ab6721	Abcam (UK)
Immunofluorescence staining—primary antibodies			
MAP2	1:1000	ab5392	Abcam (UK)
GFAP	1:1000	ab4674	Abcam (UK)
Iba-1	1:200	ab5076	Abcam (UK)
Caspase-3 active	1:300	ab2302	Abcam (UK)
Immunofluorescence staining—secondary antibodies			
Goat anti-chicken AF	1:300	ab150173	Abcam (UK)
Donkey anti-goat AF	1:500	ab150133	Abcam (UK)
Donkey anti-rabbit TR	1:300	ab6800	Abcam (UK)

### Immunofluorescence Staining

For immunofluorescence staining, a separate group of animals was used and these animals were subjected to intracardiac perfusion for 24 h following the last administration of BP-3 or the vehicle. In order to do this, deep anesthesia was induced with ketamine (80 mg/kg) and xylazine (20 mg/kg). The rats were transcardially perfused with 250 ml of saline (0.9% NaCl, 32 °C) until all of the remaining blood was removed. Next, the animals were perfused with 500 ml of cold (4 °C) 4% PFA in 0.1 M phosphate-buffered saline (PBS). The brains were removed and immersed in the same 4% PFA solution overnight. At the start of the following day, the brains were immersed in sucrose solutions in 0.1 M PBS (concentration gradient 10%, 20%, and 30%) until they sank and stored (− 80 °C) until sectioning. The tissues were cut into coronal 20-μm sections using an automatic cryostat (Leica CM1860, Germany) and placed on microscopic slides. The slides were stored at − 20 °C until staining.

Immunostaining procedure: For active caspase-3 staining, trisodium citrate buffer solution (pH ≈ 9) was used as an antigen retriever buffer. The antigen retrieval was carried out in glass containers in a water bath for 30 min at 80 °C. Next, the containers were removed from the water bath and allowed to return to room temperature. All of the sections were washed twice in 0.2% Tween in PBS solution (PBST). Depending on the combination of secondary antibodies, 10% donkey normal serum (DNS) or 10% donkey and goat normal serum (1:1) (DNS + GNS) solution in PBST was used to block nonspecific antibody binding. The tissues were incubated in blocking medium for 1 h at room temperature. Next, the blocking medium was removed, and the tissues were immersed in appropriate primary antibody solutions (anti-Caspase 3 active, 1:300, ab2302, Abcam (UK)). The tissues were double-stained together with a neuronal marker (anti-MAP2, 1:1000, ab5392, Abcam (UK)), astroglial marker (anti-GFAP, 1:1000, ab4674, Abcam (UK)) or microglial marker (anti-Iba1, 1:200, ab5076, Abcam (UK)). Each mixture of two specific antibodies was dissolved in an appropriate 2% DNS or DNS + GNS serum in PBST. The slides were incubated with primary antibody solutions at 4 °C overnight. The following day, the slides were removed and incubated for 1 h on a shaker at room temperature. Next, the sections were washed twice in 2% DNS or DNS + GNS in PBST. The tissues were immersed in appropriate secondary antibodies (goat anti-chicken AF, 1:300, ab150173, Abcam (UK); donkey anti-goat AF, 1:500, ab150133, Abcam (UK); and donkey anti-rabbit TR, 1:300, ab6800, Abcam (UK)) solution in PBST and incubated for 1 h in the dark at room temperature. The antibodies used are shown in Table 1. Afterwards, the sections were washed three times in PBST, dried, and mounted in Fluoroshield mounting medium (Sigma, USA) and coverslipped. The staining was visualized using a Leica DMI8 (Germany) fluorescence inverted microscope. Images of motor-frontal cortex, as well as CA1, CA3, and DG fields of the hippocampus, were captured by a digital CCD camera (Leica DFC450, Germany) and expressed as the relative fluorescence unit (RFU).

### Measurement of GPx Activity

GPx activity was estimated using method described by Lawrence and Burk (1976) based on the oxidation of NADPH to NADP^+^. Briefly, frozen samples of hippocampus were homogenized on ice and diluted in potassium sodium phosphate buffer (pH = 7). The working solution added to homogenates included NADPH, reduced glutathione (GSH), sodium azide, EDTA, and glutathione reductase (GR). Furthermore, hydrogen peroxide was used as a substrate, whose degradation in the presence of GSH was proceeded. GR catalyzed the NADPH-driven reduction of the generated oxidized glutathione (GSSG) followed by NADP^+^ formation. The decrease in samples’ absorbance proportional to the activity of GPx was measured at 340 nm for 30 min at 37 °C by multiwell plate reader POLARstar Omega (BMG Labtech, Germany). Results were expressed relative to the total amount of protein in samples using the BCA Protein Assay Kit (Thermo, USA).

### Determination of Blood Hormone Levels

The concentration of 17β-estradiol (ab108667; Abcam, UK), progesterone (CSB-E07282r; Cusabio, China), testosterone (ab108666; Abcam, UK), prolactin (CSB-E06881r; Cusabio, China), fT3 (DKO037; DiaMetra, Italy), fT4 (DKO038; DiaMetra, Italy), and thyroid-stimulating hormone (TSH; CSB-E05115r; Cusabio, China) were determined using commercial ELISA kits. The assays were performed using undiluted plasma according to the manufacturers’ manuals. The absorbance was measured in a multiwell plate reader (TECAN Infinite M200 PRO), and hormone concentrations in the samples were calculated from a calibration curve.

### Hematological Analyses

Immediately after collection, whole blood samples with heparin were used for hematological analyses. The leukocyte count (WBC), red blood cell count (RBC), platelet count (PLT), hemoglobin concentration (HGB), hematocrit (HCT), mean corpuscular volume (MCV), mean cell hemoglobin (MCH), and mean cell hemoglobin concentration (MCHC) were analyzed by means of a Cobas Micros (Roche, Palo Alto, CA, USA) analyzer. Hematological analyses were systematically checked as reported previously (Starek et al. 2008).

### Statistical Analysis

All data were expressed as the means (±SEM—standard error of the mean) and analyzed in a GraphPad Prism (version 6.0, USA) program. Normal distribution of data sets was determined by a Shapiro-Wilk normality test, and *t* tests were used in further statistical analysis. The *p* value below 0.05 was considered statistically significant.

## Results

### The Effect of BP-3 on Short-Term and Spatial Memory in Female Rats

**Fig. 1:**
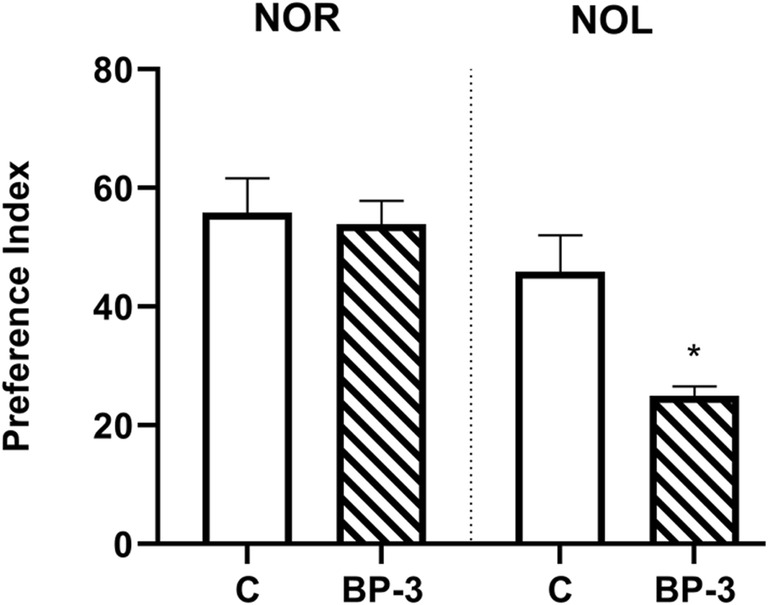
Effects of BP-3 dermal administration to female rats on short-term memory performance in a novel object recognition test (NOR) and spatial memory performance in a novel object location test (NOL) (*n* = 7). The results are expressed as the means of the preference index ± S.E.M. * *p* < 0.05 vs control group; BP-3—benzophenone-3; C—control group

The short-term and spatial memories were determined in a new object and new location recognition test, respectively, and presented as the preference index (Fig. 1). Exposure of female rats to BP-3 in prenatal and, next, at 2 weeks of adulthood had no effect on the short-term memory (*p* = 0.8127), but significantly disturbed the spatial memory (*p* < 0.05).

### Concentration of BP-3 in Plasma, Liver, and Brain Structures and BP-1 in Liver in Female Rats

**Fig. 2:**
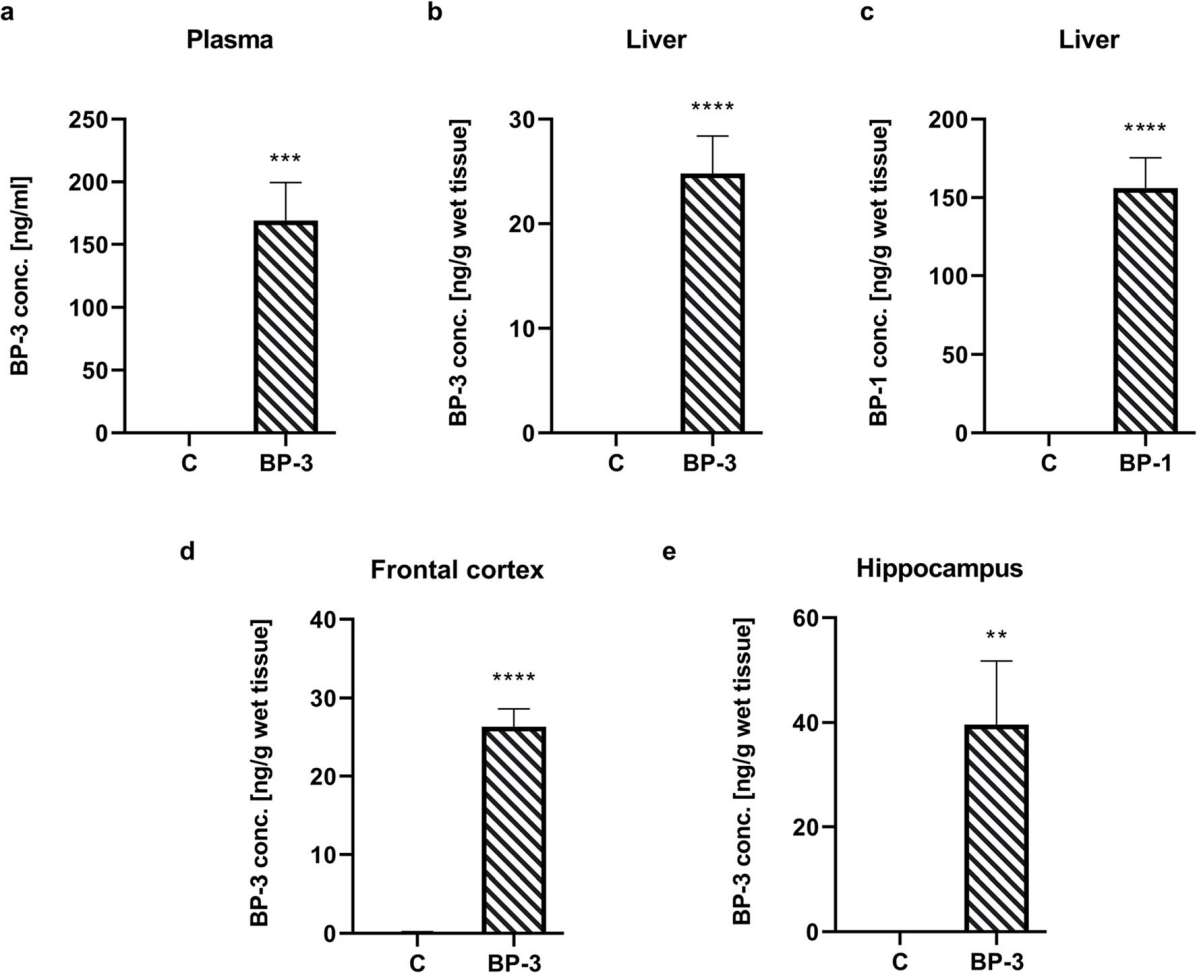
Concentration of BP-3 in plasma (**a**); liver (**b**); frontal cortex (**d**); hippocampus (**e**), and BP-1 content in liver (**c**) (*n* = 6). All values are expressed as the means *±* S.E.M*.* **p < 0.01, ***p < 0.001, ****p < 0.0001 vs control group. BP-1—benzophenone-1; BP-3—benzophenone-3; C—control group

BP-3 levels were determined 24 h after the last administration of this compound. In the plasma of all of the control animals, the level of BP-3 was below the limit of detection, while in animals exposed to this compound it was in the range of 70–220 ng/ml (average 169 ng/ml) (Fig. 2a) (*p* < 0.001). In the liver, a much higher level of the main BP-3 metabolite, BP-1 (156 ng/g wet tissue), was found, while the parent compound occurred in 25 ng/g wet tissue concentration (Fig. 2b, c) (*p* < 0.0001 and *p* < 0.0001). In BP-3-treated animals, this compound reached a concentration of 26 ng/g in the frontal cortex (*p* < 0.0001) and 40 ng/g in the hippocampus (*p* < 0.01), whereas in any of these brain structures the presence of BP-1 was found (Fig. 2d, e). In the control group, the BP-3 level in the hippocampus was above the detection limit in only one female, while it was not in the frontal cortex and liver.

### Total Antioxidant Activity and Lipid Peroxidation in Brain Structures of Female Rats Exposed to BP-3

**Fig. 3:**
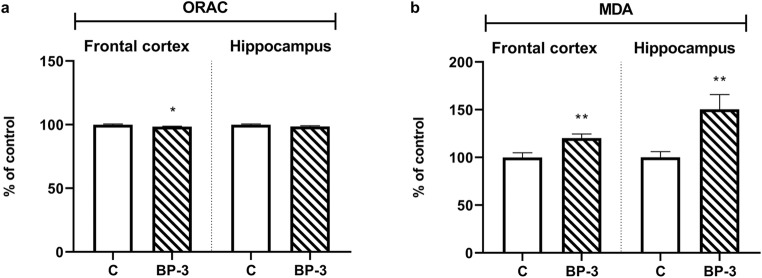
Effects of BP-3 exposure on antioxidant capacity in the frontal cortex and hippocampus (**a**) and lipid peroxidation levels in these brain structures (**b**) (*n* = 8). The data were recalculated and expressed as a percent of the control ± S.E.M, where 100% is the basal antioxidant capacity and lipid peroxidation in control animals. *p < 0.05, **p < 0.01 vs control group. BP-3—benzophenone-3; C—control group

It has been found that BP-3 did not change the antioxidant activity of hippocampal tissue samples (*p* = 0.1195), while to a small extent yet significantly it decreased this parameter in frontal cortex (*p* < 0.05) (Fig. 3a). However, the tested compound significantly and relatively strongly increased lipid peroxidation in the hippocampus (*p* < 0.01) and, especially, in the frontal cortex (*p* = 0.01) (Fig. 3b).

### The Effect of BP-3 on the Extracellular Glutamate Concentrations and Expression of Main Glutamate Transporters in the Hippocampus and Frontal Cortex

**Fig. 4:**
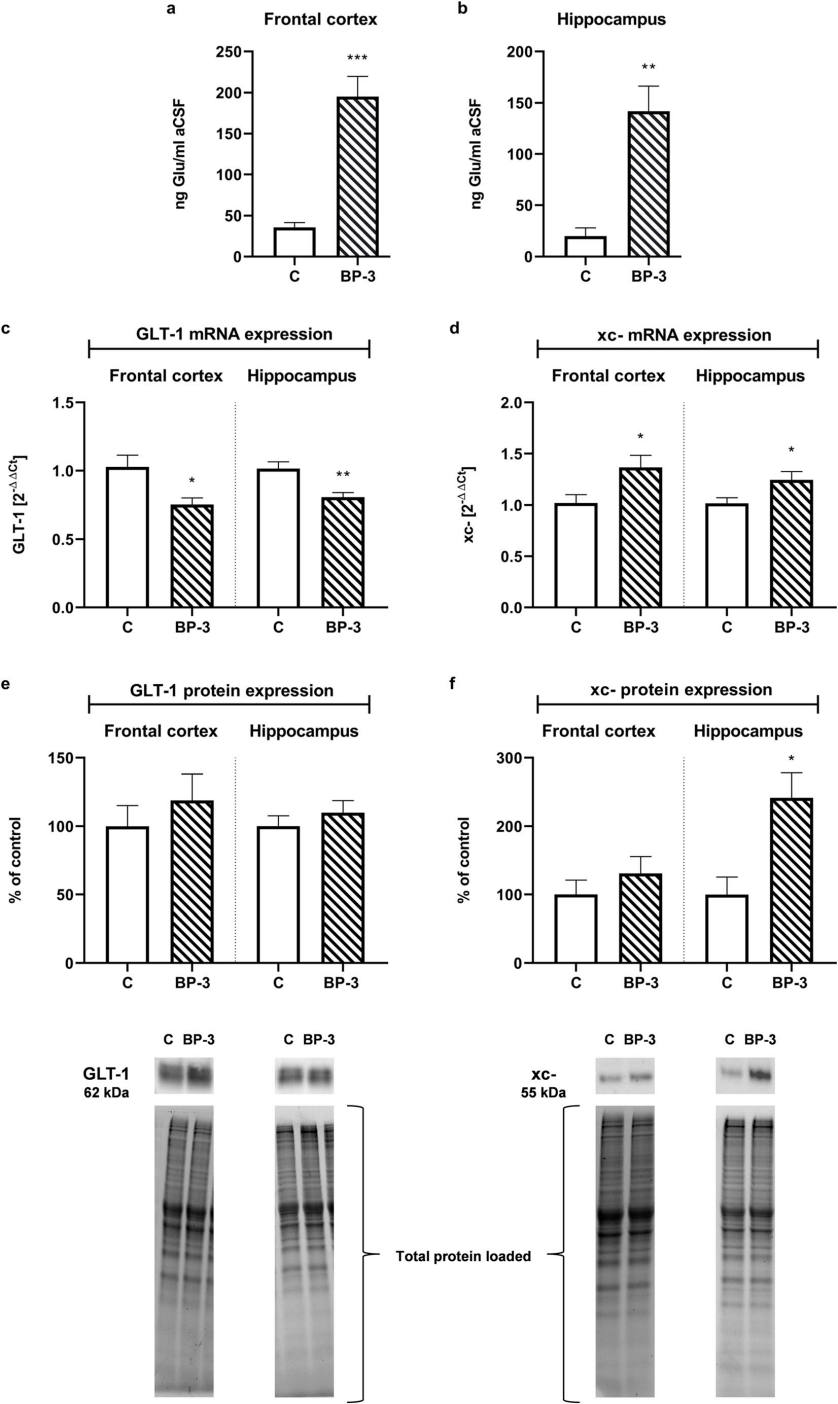
Effects of BP-3 dermal administration on the concentration of Glu in the extracellular space in the frontal cortex (**a**) and hippocampus (**b**), on the mRNA expression of the Glu transporters GLT-1 (**c**) and xCT (**d**) and protein levels of GLT-1 (**e**) and xCT (**f**) in the frontal cortex and hippocampus (*n* = 7). All the values are shown as the means ± S.E.M. *p < 0.05, **p < 0.01, ***p < 0.001 vs control group. aCSF—artificial cerebrospinal fluid; BP-3—benzophenone-3; C—control group. At the bottom representative immunoblots showing the expression of GLT-1 and xCT in the frontal cortex and hippocampus with reference to the total protein concentration loaded on the gel were shown

The extracellular glutamate concentration in the control animals was higher in the frontal cortex (*p* < 0.001) than in the hippocampus (*p* < 0.01) (Fig. 4a, b). Exposure to BP-3 increased the level of this neurotransmitter by 7.5-fold in the hippocampus and by 5.8-fold in the frontal cortex.

Determining the expression of the mRNA and protein levels of the two major glutamate transporters illustrated that BP-3 significantly decreased GLT-1 mRNA (frontal cortex *p* < 0.05, hippocampus *p* < 0.01) and increased xc-mRNA (frontal cortex *p* < 0.05, hippocampus *p* < 0.05) in both of the examined brain structures, although changes in mRNA did not correlate with the protein levels (Fig. 4c–f). In the frontal cortex, BP-3 had no effect on GLT-1 (*p* = 0.4568) and xc- (*p* = 0.3633) transporters, while in the hippocampus the GLT-1 protein level also did not change (*p* = 0.4222), but significantly and strongly increased the xc- transporter (p < 0.05).

### The Effect of BP-3 on Estrogen and Progesterone Receptors’ Expression in the Hippocampus and Frontal Cortex

**Fig. 5:**
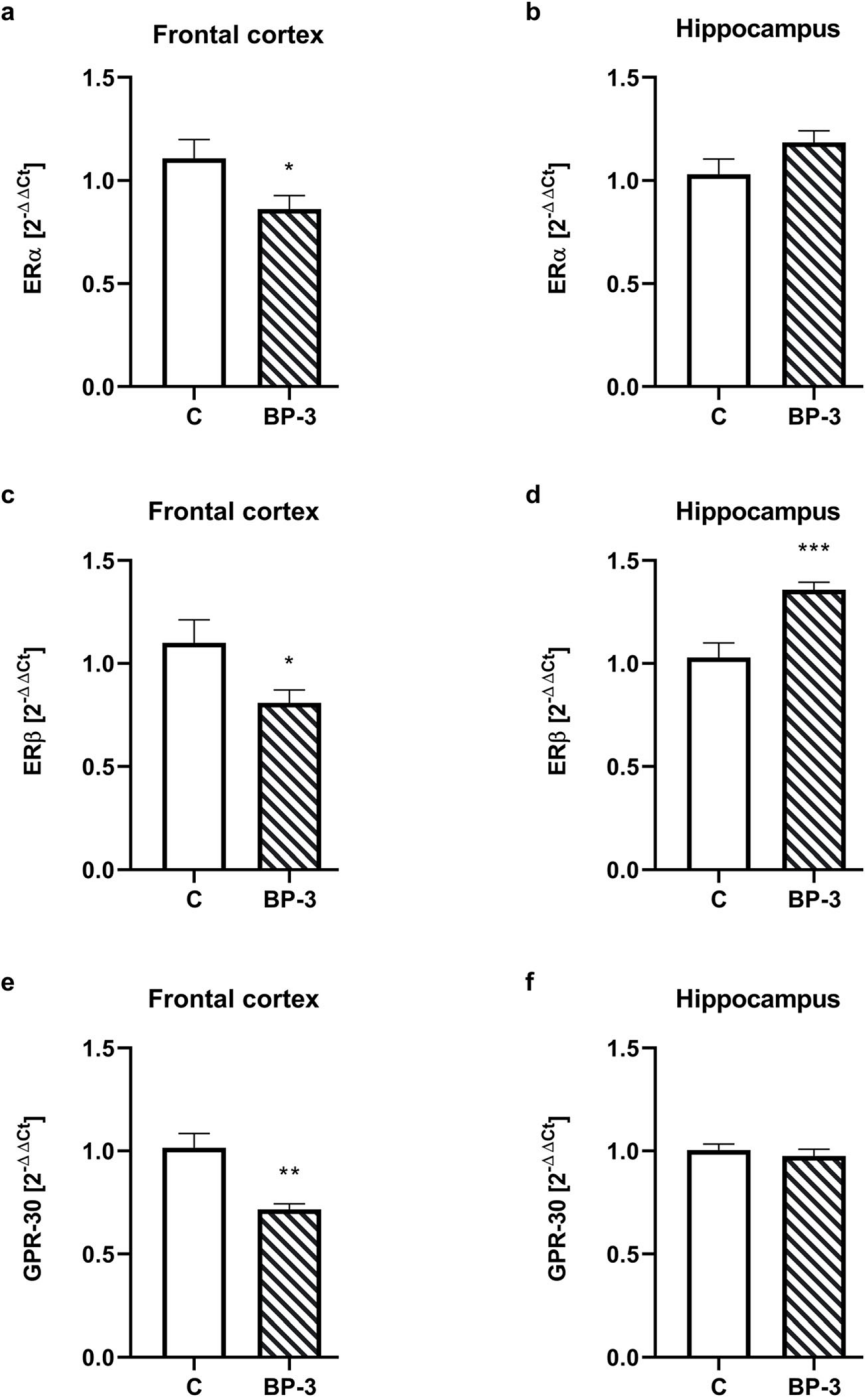
Effects of BP-3 exposure on the mRNA expression of the estrogen receptors: ERα (**a**, **b**), ERβ (**c**, **d**) and GPR-30 (**e**, **f**) in the frontal cortex and hippocampus (*n* = 8). All the values are shown as the means ± S.E.M. *p < 0.05, **p < 0.01, ***p < 0.001 vs control group. BP-3—benzophenone-3; C—control group

In the frontal cortex, mRNA coding of all three types of ER, i.e., ERα, ERβ, and GPR-30, was significantly reduced (*p* < 0.05, *p* < 0.05, *p* < 0.01, respectively) in the female rats exposed to BP-3 (Fig. 5a–e). In the hippocampus, there were no differences in the mRNA of ERα (*p* = 0.1161) and GPR-30 (*p* = 0.5084) receptors, while mRNA ERβ was significantly higher in BP-3-treated rats than in the control animals (*p* < 0.001) (Fig. 5b–f). At the protein level, there were no significant differences in the levels of ERα assayed in both the cytosolic and nuclear fraction, in the nuclear ERβ concentration, and in the membrane GPR-30 receptor in the frontal cortex and hippocampus between animals receiving BP-3 and controls (Table 2). The PR concentration did not change in the frontal cortex, while BP-3 reduced its level in the cytosol fraction of the hippocampus.

**Table 2:** Effects of BP-3 dermal administration to female rats on the level of estrogen (ERα, ERβ, and GRP30) and progesterone receptors (PR) in the frontal cortex and hippocampus

Protein	Fraction	Brain structure	C	BP-3	*p*
ERα	Cytosol	Frontal cortex	100.0 ± 3639	96.50 ± 11.62	0.7938
		Hippocampus	100.0 ± 22.74	122.0 ± 30.48	0.5734
	Nucleus	Frontal cortex	100.0 ± 25.61	101.6 ± 34.16	0.9708
		Hippocampus	100.0 ± 27.02	75.04 ± 17.06	0.4529
ERβ	Nucleus	Frontal cortex	100.0 ± 13.27	101.4 ± 7260	0.9285
		Hippocampus	100.0 ± 8386	102.3 ± 7092	0.8401
GPR30	Membrane	Frontal cortex	100.0 ± 9807	113.5 ± 9224	0.3362
		Hippocampus	100.0 ± 7361	108.0 ± 6389	0.4253
PR	Cytosol	Frontal cortex	100.0 ± 6363	94.44 ± 5378	0.5175
		Hippocampus	100.0 ± 7013	79.74 ± 3992	0.0355
	Nucleus	Frontal cortex	100.0 ± 6032	114.1 ± 11.24	0.2918
		Hippocampus	100.0 ± 16.60	95.10 ± 8147	0.8066

### The Effect of BP-3 on the Expression of AhR in Brain Structures of Female Rats

**Fig. 6:**
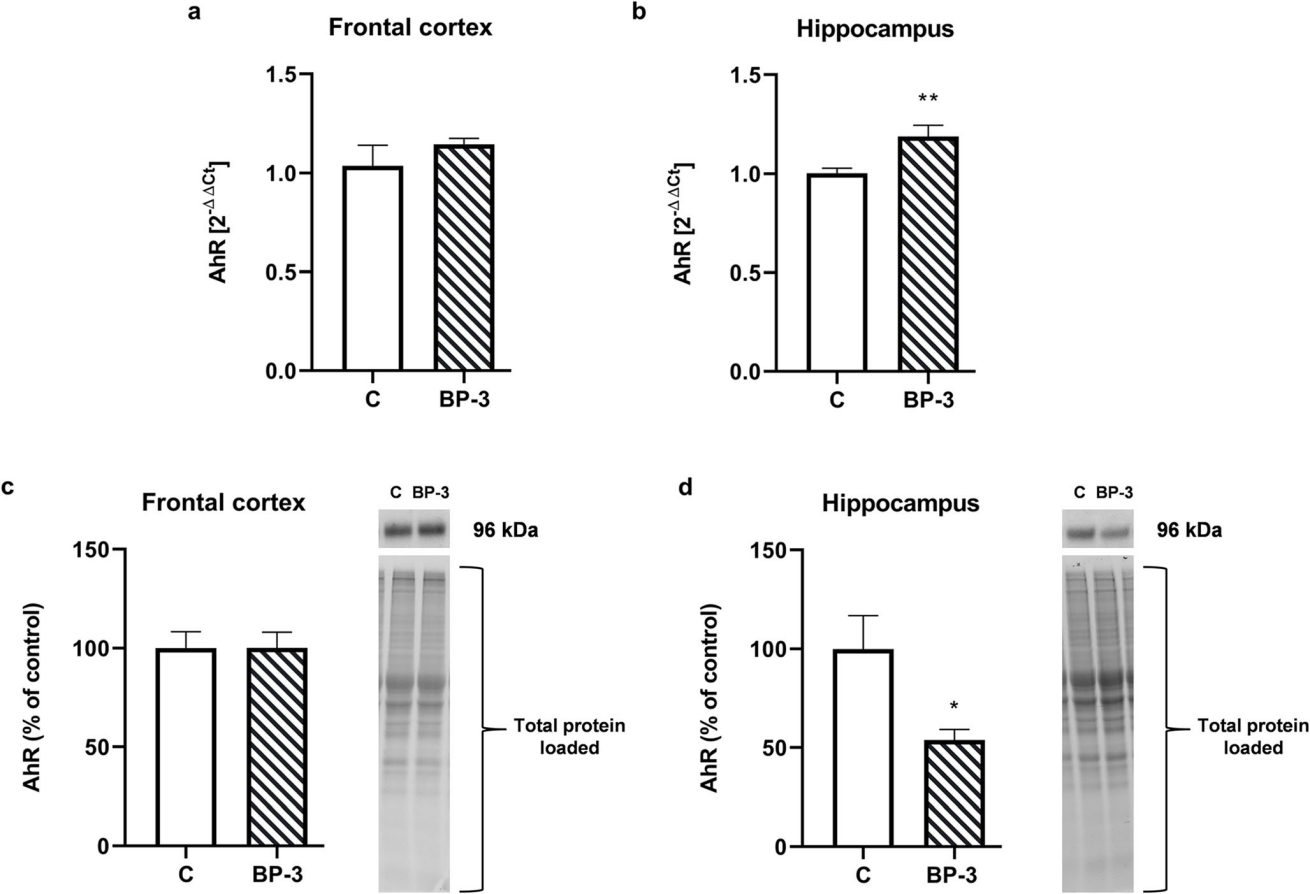
Effects of BP-3 exposure on the mRNA expression of AhR in the frontal cortex (**a**) and hippocampus (**b**) and on the protein level of AhR in the frontal cortex (**c**) and hippocampus (**d**) (*n* = 8). All the values are shown as the means ± S.E.M. **p < 0.01 vs control group. BP-3—benzophenone-3; C—control group. Representative immunoblots showing the expression of AhR in the frontal cortex (**c**) and hippocampus (**d**) with reference to the total protein concentration loaded on the gel were shown

In the frontal cortex, BP-3 had no effect on the AhR expression on both the mRNA (*p* = 0.3323) and protein (*p* = 0.9853) level (Fig. 6a, c), although in the hippocampus of BP-3-treated animals a significant increase in mRNA expression (*p* < 0.01) and a decrease in the protein (*p* < 0.05) level of AhR were observed (Fig. 6b, d).

### Lack of BP-3 Effect on Apoptotic Markers in the Hippocampus and Frontal Cortex

It has been shown that BP-3 had no effect on the concentration of the active form of caspase-3, the executive enzyme in the apoptotic process, as well as on the key enzymes in the intracellular (caspase-9) and receptors’ (caspase-8) apoptotic pathway in the hippocampus and frontal cortex (Table 3). Also, the test compound did not change the level of the main pro-apoptotic (Bax) and anti-apoptotic (Bcl-2) proteins in both of the examined brain structures.

**Table 3:** Effects of BP-3 dermal administration to female rats on selected markers of apoptosis in the frontal cortex and hippocampus

Protein	Fraction	Brain structure	C	BP-3	*p*
Caspase-3 active	Cytosol	Frontal cortex	100.0 ± 12.31	100.9 ± 16.78	0.9664
		Hippocampus	100.0 ± 24.43	113.8 ± 21.06	0.6852
Caspase-8 active	Cytosol	Frontal cortex	100.0 ± 26.32	123.7 ± 63.00	0.7358
		Hippocampus	100.0 ± 23.34	87.81 ± 22.88	0.7156
Caspase-9	Cytosol	Frontal cortex	100.0 ± 6028	116.3 ± 5682	0.0731
		Hippocampus	100.0 ± 12.95	107.5 ± 12.51	0.6861
Bax	Cytosol	Frontal cortex	100.0 ± 11.18	104.9 ± 20.35	0.8379
		Hippocampus	100.0 ± 22.77	75.53 ± 14.40	0.3817
Bcl-2	Membrane	Frontal cortex	100.0 ± 10.19	104.9 ± 16.79	0.8077
		Hippocampus	100.0 ± 11.87	121.7 ± 22.84	0.4147

**Fig. 7:**
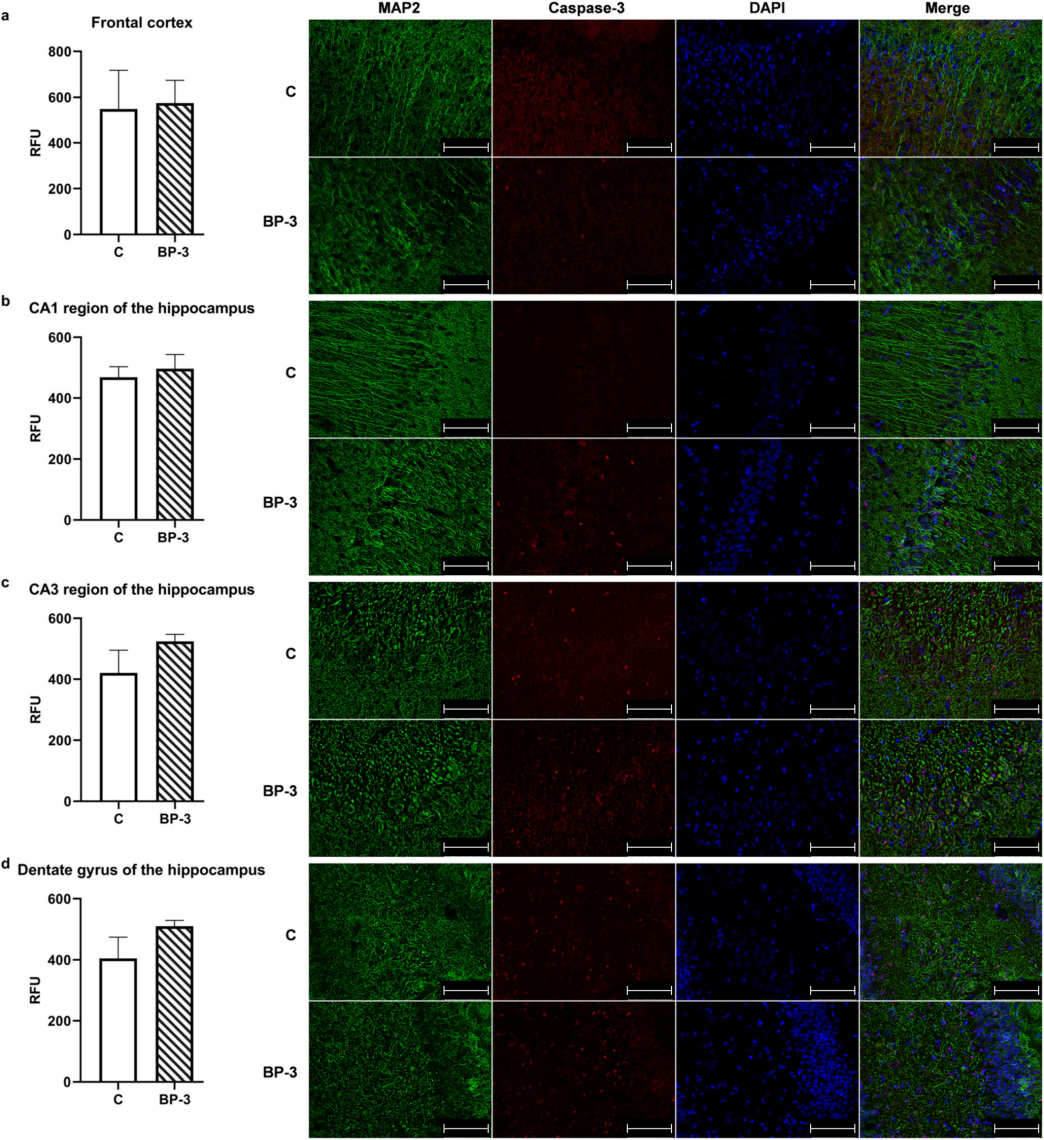
Effects of BP-3 on the relative expression of the active form of caspase-3 in neurons of the frontal cortex (**a**), the CA1 region of the hippocampus (**b**), the CA3 region of the hippocampus (**c**) and the dentate gyrus (DG) of the hippocampus (**d**) (*n* = 4). The red (TexasRed) fluorescence data are expressed as the relative fluorescence unit (RFU) ± S.E.M. Representative microphotographs for the frontal cortex and the CA1, CA3, and DG regions of the hippocampus have been placed. Caspase-3 (TexasRed, red), MAP2 (FITC, green), and nucleus (DAPI, blue) staining of 20-μm-thick brain sections of control animals and animals exposed to BP-3. Scalebar = 75 μm

The lack of BP-3 influence on caspase-3 activation has also been confirmed by immunofluorescence studies, in which a similar expression of the active form of this enzyme in neurons (labeled with MAP-2) was demonstrated in the frontal cortex (*p* = 0.8921) and CA1 (*p* = 0.6693), CA3 (*p* = 0.1475), and dentate gyrus (DG) (*p* = 0.1123) regions of the hippocampus in control and BP-3 treated animals (Fig. 7).

### Lack of BP-3 Effect on Microglia Activation

Exposure of animals to BP-3 had no effect on mRNA expression of selected markers of microglia cells’ activation, i.e., *Aif*, a microglia/macrophage-specific calcium-binding protein, *Cd40*, a costimulatory receptor occurring on the antigen-presenting cells, and *C1q*, a initiating protein in the classical pathway of complement activation (data not shown). Also, the Iba1 factor determined in the hippocampus and frontal cortex at the protein level was at a similar concentration in control and BP-3-treated animals (data not shown).

### Lack of BP-3 Effect on Caspase-1, RIP-1 Protein and GPx in the Hippocampus

**Fig. 8:**
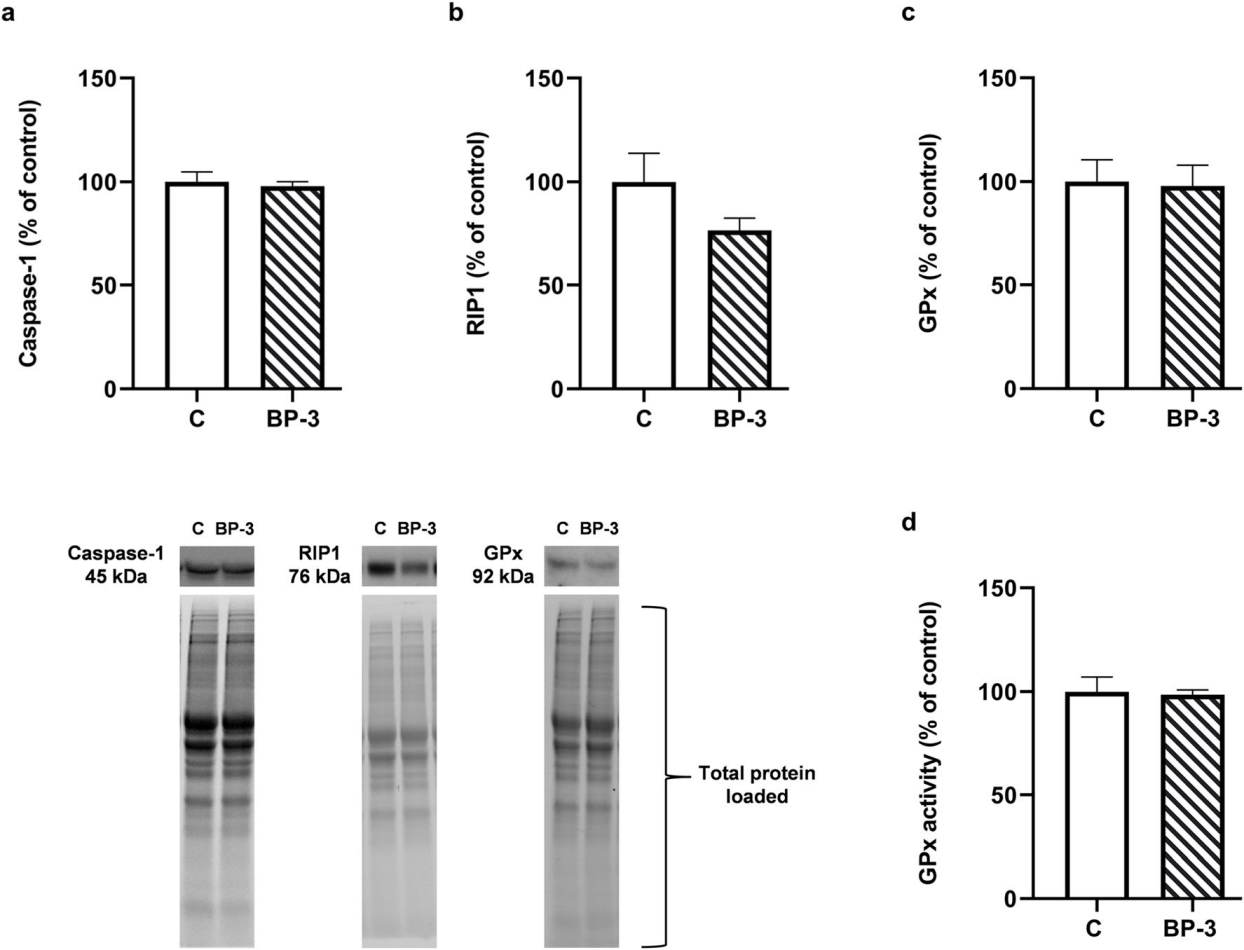
Effects of BP-3 exposure on the protein expression of active form of caspase-1 (**a**), threonine-serine kinase RIP-1 (**b**), glutathione peroxidase (**c**), and activity of GPx (**d**) in the hippocampus (*n* = 7). All the values are shown as the means ± S.E.M. RIP-1—threonine-serine kinase RIP-1; GPx—glutathione peroxidase; BP-3—benzophenone-3; C—control group. Representative immunoblots showing the expression of caspase-1, RIP1, and GPx in the hippocampus with reference to the total protein concentration loaded on the gel were shown

Because there was no activation of apoptosis in the hippocampus and frontal cortex of animals exposed to BP-3, yet an increase in the glutamate level and lipid peroxidation were observed, selected markers of regulated necrosis, i.e., necroptosis and pyroptosis, were therefore determined in the hippocampus, a structure more vulnerable to damage than the frontal cortex. However, it was found that BP-3 had no effect on the caspase-1 (*p* = 0.7074) and RIP-1 (*p* = 0.1277) protein level, as well as on the expression and activation of glutathione peroxidase (*p* = 0.8924, *p* = 0.8358, respectively) in this structure (Fig. 8a–d).

### The Effect of BP-3 on the Sex and Thyroid Hormones Plasma Concentration

**Fig. 9:**
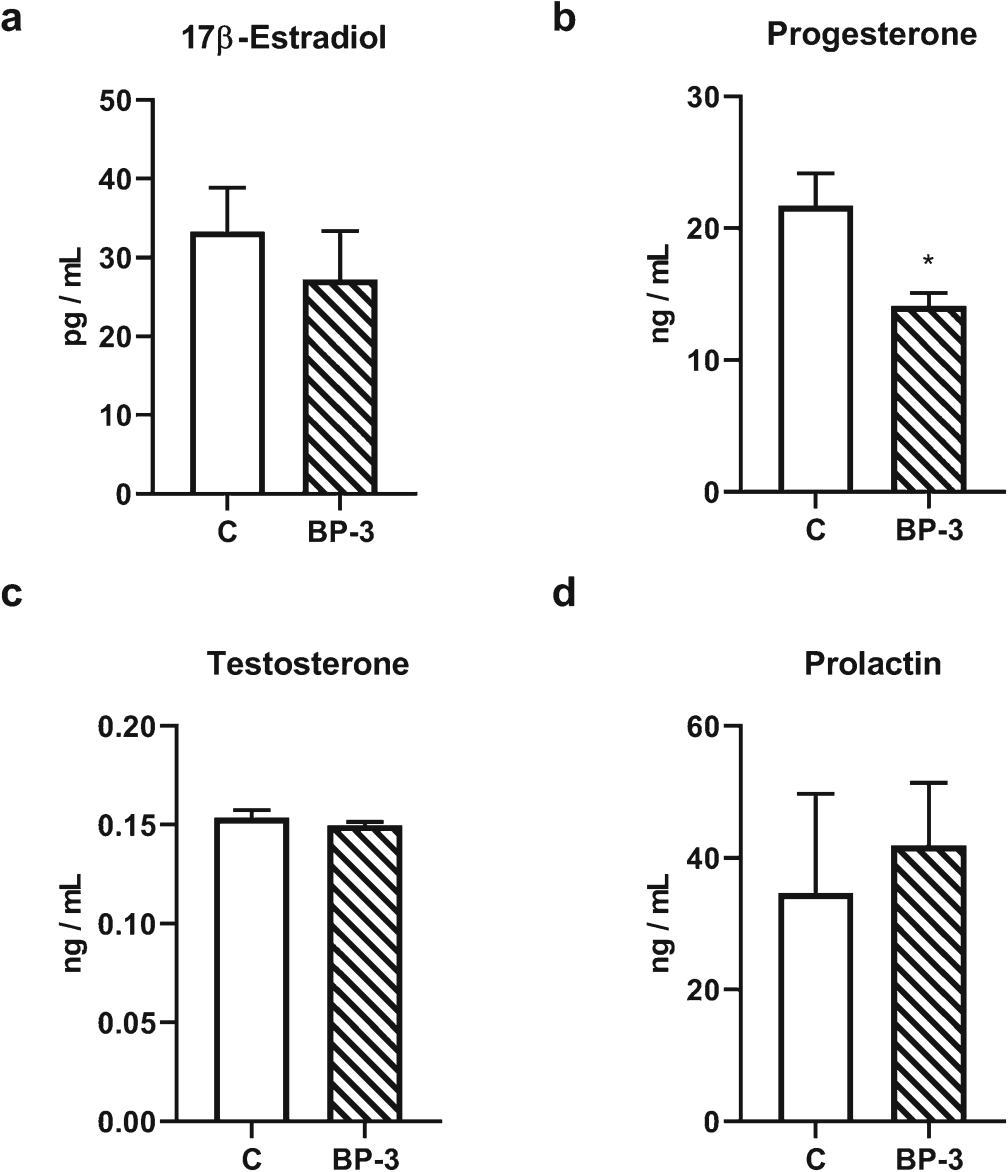
Effects of BP-3 exposure on 17β-estradiol (**a**), progesterone (**b**), testosterone (**c**), and prolactin (**d**) plasma level (*n* = 7). All data are expressed as the means ±SEM. *p < 0.05 vs control group. BP-3—benzophenone-3; C—control group

Exposure of female rats to BP-3 during the prenatal period and, next, for 2 weeks into adulthood had no significant effect on the blood 17β-estradiol (*p* = 0.4871), testosterone (*p* = 0.3840), and prolactin (*p* = 0.7139) levels, but significantly decreased the concentration of progesterone (*p* < 0.05) (Fig. 9a–d).

**Fig. 10:**
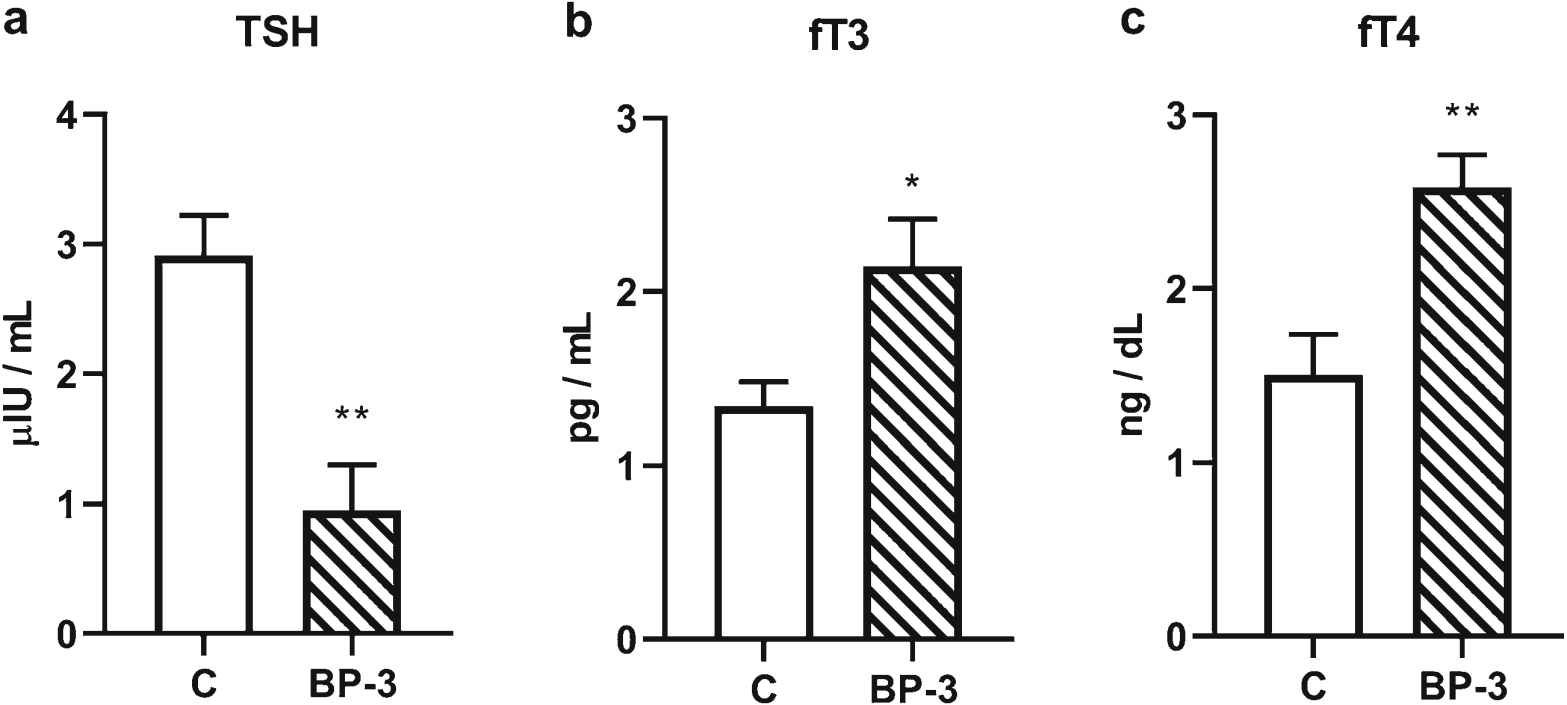
Effects of BP-3 administration on TSH (**a**), fT3 (**b**), and fT4 (**c**) plasma concentration (*n* = 7). All data are expressed as the means ± SEM. TSH—thyroid-stimulating hormone; fT3—free fractions of triiodothyronine; fT4—free fractions of thyroxin; *p < 0.05, **p < 0.01 vs control group. BP-3—benzophenone-3; C—control group

Statistically significant higher levels of a free, unbound, active form of thyroid hormones, both fT3 (*p* < 0.05) and fT4 (*p* < 0.01), were found in the blood of female rats administered BP-3 (Fig. 10b, c). The hyperthyroidism induced by BP-3 was also confirmed by significantly lower TSH concentration in animals receiving this compound than in control animals (p < 0.01) (Fig. 10a).

### The Effect of BP-3 on the Hematological Parameters

**Fig. 11:**
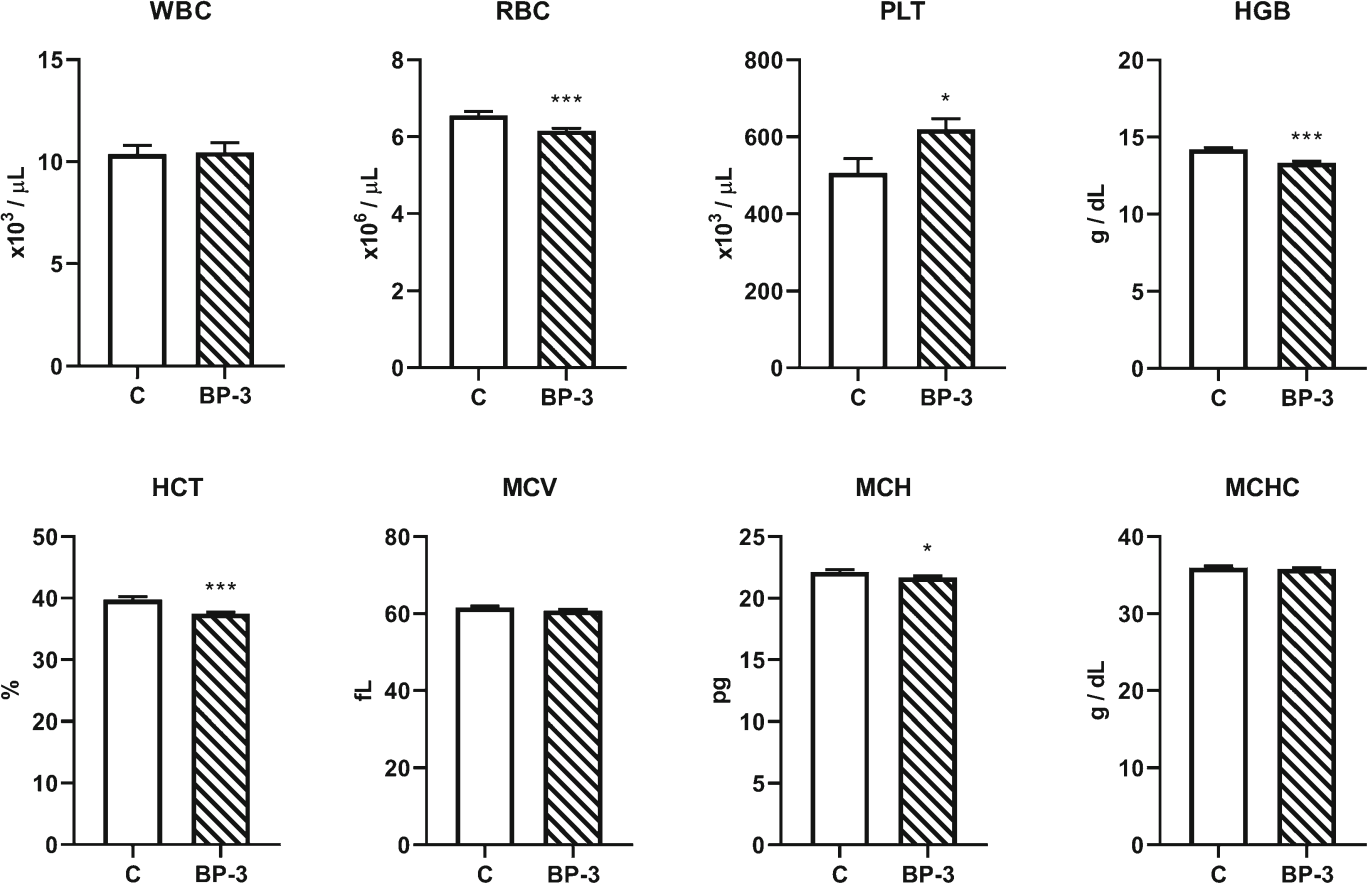
The effect of benzophenone-3 administration on hematological parameters in female rat blood (*n* = 12). All data are expressed as the means ± SEM. WBC—leukocyte count; RBC—red blood cell count; PLT—platelet count; HGB—hemoglobin concentration; HCT—hematocrit; MCV—mean corpuscular volume; MCH—mean cell hemoglobin; MCHC—mean cell hemoglobin concentration; BP-3—benzophenone-3; C—control group

It has been found that BP-3 decreased the red blood cell (RBC) count (*p* < 0.001), hemoglobin concentration (HGB) (*p* < 0.001), hematocrit (HCT) (*p* < 0.001), and corpuscular hemoglobin concentration (MCH) (*p* < 0.05), and increased the platelet count (PLT) (*p* < 0.05), but had no effect on the leukocyte count (WBC) (*p* = 0.8792), with the mean corpuscular volume (MCV) (*p* = 0.1169) and mean cell hemoglobin concentration (MCHC) (*p* = 0.6108) (Fig. 11).

## Discussion

Current studies have shown that after the same exposure to BP-3, this compound was present in the blood of females at a lower concentration than that observed in our previous studies in males (Pomierny et al. 2019). This is consistent with data showing that after dermal application of BP-3, its maximum plasma and urine concentrations in humans are higher in men than in women (Janjua et al. 2008). Moreover, in the present study, the blood BP-3 level in female rats was similar to that observed in humans after 4 days of whole-body topical application of 2 mg/cm^2^ of a sunscreen formula containing 10% BP-3 (Janjua et al. 2008). Also, the level of BP-3 in the liver in female rats was significantly lower than that previously observed in males, while the levels of its main metabolite, BP-1, were comparable. The liver is the main organ for BP-3 biotransformation (Kim and Choi 2014; Okereke et al. 1993) and the higher ratio of the metabolite to the parent compound observed in this tissue in female rats, rather than males, indicated that females probably metabolize this compound faster.

Some data show that BP-3 not only is easily absorbed through the skin but also passes through the blood-brain barrier (Krzyżanowska et al. 2018; Wnuk et al. 2018b). However, the key issue is whether this compound can achieve sufficient concentration in the brain structures to affect the function or reduce the survival of nerve cells. Neurodegenerative effects of BP-3 have been relatively well demonstrated in vitro (Broniowska et al. 2016; Wnuk et al. 2018a), but in vivo only after prenatal exposure of mice to this compound (Wnuk et al. 2018b, 2019), and in male rats after combined prenatal and adult exposure (Pomierny et al. 2019). In the BP-3 exposure model we used, the BP-3 concentration in the frontal cortex and hippocampus of male rats was about 50 ng/g, while in the hippocampus of female rats it was about 40 ng/ml, although in the frontal cortex it was only 26 ng/ml. A higher concentration of BP-3 in the hippocampus than in the frontal cortex of female rats may be the cause of a greater increase of lipid peroxidation, which is an important marker of free radical action on the membrane polyunsaturated fatty acids observed in this structure.

The neurotoxic effect of some xenobiotics result from changes in the brain expression of estrogen receptors, especially a decrease in nuclear ERβ and membrane GPR30 and, in consequence, inhibition of estrogen’s neuroprotective action (Lan et al. 2014; Lebesgue et al. 2009; Lee et al. 2012; Zhang et al., 2018). In the present study, a decrease in the mRNA level of all ER subtypes in the frontal cortex was observed, but there were no changes at the protein level either in the frontal cortex or in the hippocampus. Moreover, the 17β-estradiol blood concentration also did not change in the female rats exposed to BP-3. In contrast to results obtained in female rats, we previously observed a decrease in nuclear ERβ and membrane GPR30 levels in the hippocampus and frontal cortex, and ERα in the hippocampus of male rats, thus indicating gender-dependent BP-3 action. The lack of the BP-3 effect on ER expression in female rats may result from differences in the concentration of this compound in the brain or from various ER expressions in female and male rats.

In contrast to the lack of the BP-3 effect on the ER level in the brain of female rats, the effect of this compound on the concentration of extracellular glutamate in the hippocampus and frontal cortex in females was similar to that of the previously observed males. Moreover, despite a significantly higher BP-3 concentration in the hippocampus than in the frontal cortex in both of the studied brain structures, BP-3 with a similar potency increased the extracellular glutamate level. Enhanced release of glutamate and, in consequence, excessive stimulation of NMDA receptors and an increase in intracellular Ca^2+^ is considered to be the most important cause of neuronal degeneration (Brassai et al. 2015; Lau and Tymianski 2010). The concentration of glutamate in the synapse is mainly regulated by the uptake of this neurotransmitter into glial cells, primarily into astrocytes, by the plasma membrane GLT-1 transporter. BP-3 decreased mRNA coding GLT-1 in the hippocampus and frontal cortex, although this change did not translate to the GLT-1 protein level. However, the second essential transporter in regulating the extracellular glutamate level, xCT, could be responsible for the increased level of this neurotransmitter in the hippocampus. xCT is a cystine/glutamate antiporter which exchanges extracellular cysteine for intracellular glutamate, and consequently, it increases the level of extracellular glutamate (Massie et al. 2015). We found that BP-3 increased the mRNA of this transporter in both brain structures studied, and strongly raised the level of xCT protein in the hippocampus, yet had no effect on its concentration in the frontal cortex. Thus, unlike in males, in females the increase in extracellular glutamate concentration correlates well only with changes in xCT, yet not GLT-1, and only in the hippocampus. The cause of the effect of BP-3 on the level of glutamate in the frontal cortex of female rats is difficult to explain, because it was not due to changes in the examined glutamate transporters or, indeed, also not connected with microglial activation, since this compound did not increase the expression of the tested microglia markers.

The obtained results also indicate that, although BP-3 increased glutamate levels and intensified lipid peroxidation in both brain structures, it did not induce the process of apoptosis. This was an unexpected result as enhanced lipid peroxidation, one of the effects of free radical action on membrane polyunsaturated fatty acids, argued that BP-3 induced oxidative stress, which has often evoked the intracellular pathway of apoptosis. The brain, due to weak antioxidant activity and strong production of free radicals during mitochondrial respiration, and the metabolism of some neurotransmitters, mainly dopamine, is particularly sensitive to oxidative stress-induced damage. We have previously observed changes in apoptosis markers in males exposed to BP-3 in the frontal cortex, whereas in the hippocampus, increased caspase-3 activity had only been demonstrated in the CA1 region by fluorescent staining, yet not when determined by Western blotting (Pomierny et al. 2019). The lack of signals for the induction of apoptosis in females may have resulted from a lower BP-3 concentration in the brain of females, especially in the frontal cortex, than in males, which has not yet led to the death of neurons. However, it cannot be ruled out that BP-3 induced the death of neurons by other means than apoptosis, such as necroptosis, pyroptosis or ferroptosis, which are types of programmed necrosis, which occurs without chromatin condensation and caspase-3, 8, and 9 activation. This possibility was studied in the hippocampus, because in this structure the concentration of BP-3 was about 60% higher than in the frontal cortex, and in this tissue lipid peroxidation was also more severe. The lack of changes in the level of the active form of caspase-1 and expression of threonine-serine kinase—RIP1, markers of pyroptosis and necroptosis, respectively revealed that induction of these processes by BP-3 was rather unlikely (Chen et al. 2016). Likewise, no reduction in glutathione peroxidase expression and activity in the hippocampus suggested that BP-3 also did not induce the ferroptosis process (Xie et al. 2016).

The results obtained suggest that the adverse changes observed in the female rats exposed to BP-3, like the enhanced glutamate level and increased lipid peroxidation, did not lead to cell death. Despite the lack of apoptosis, the fact that BP-3 disturbed memory processes indicates its unfavorable effect on brain function. Indeed, we found that BP-3 did not affect short-term memory, as determined in the NOR test, but significantly inhibited spatial memory assayed by the NOL test. Since the peripheral cortex is mainly involved in the performance of the NOR test, while the hippocampus participates in the NOL test, this confirmed the biochemical results that BP-3 more strongly affects the hippocampus than the frontal cortex in female rats.

In contrast to the weaker effects of the BP-3 on females than male brains, this compound more strongly affected the endocrine system in female rats and evoked disturbance in hematological parameters. As demonstrated in the current studies, the higher levels of free, unbound, and active forms of thyroid hormones, both fT3 and fT4, while reducing TSH concentration, indicated that BP-3 caused hyperthyroidism. Most of the existing data indicates that the benzophenones rather reduce the level of thyroid hormones. For example, in epidemiological studies an association between urinary BP-3 concentrations and decreases in thyroid hormones in the general population was demonstrated (Kim et al. 2017). However, in pregnant women a positive association between serum concentrations of 4-hydroxy-benzophenone and T3 and T4 levels was observed, and previously we found that benzophenone-2 administered dermally to adult male rats also evoked hyperthyroidism (Broniowska et al. 2018; Krause et al. 2018). Increased thyroid hormone levels, which in our BP-3 administration model occur only in females yet not in males, in addition to the potential, adverse metabolic effects, may also be one of the causes of smaller changes observed in the brain of female rather than male rats through its exertion of neuroprotective effects. These hormones cross the blood-brain barrier and current data indicate that their central action occurs not only in the developmental period, but also in adults and that they exert a neuroprotective effect (Remaud et al. 2014; Vallortigara et al. 2009).

Another important effect of BP-3 in females was a strong decrease in blood progesterone levels. No change in 17β-estradiol levels and a decrease in progesterone production indicated that BP-3 mainly disturbed the function of the corpus luteum and not the ovarian follicles. However, BP-3 are known to exert the agonistic effect of ER and possibly also antagonistic on PR, so all these effects can contribute to a weakening of fertility (Schlumpf et al. 2004; Schreurs et al. 2005).

The present study also showed that BP-3 administration decreased the number of erythrocytes and hemoglobin concentration, but increased the platelet count. These were adverse effects, which suggested that BP-3 may affect the bone marrow, leading to anemia with an increase in platelets or act directly, damaging erythrocytes. The effect of BP-3 on hematological parameters has not been studied so far, but it was demonstrated that the plant benzophenone, garcinol, induced apoptosis of human erythrocytes in in vitro conditions (Fazio et al., 2015).Thus, it is possible that the reduction in erythrocytes shown in the present study was also due to the direct effect of BP-3 on red cells.

## Conclusion

In summary, the presented data indicated that after the same exposure to BP-3, in female rats, concentration of this compound in blood and in the brain was lower than previously observed in male rats. Moreover, in contrast to male rats in the frontal cortex and hippocampus of female rats’ apoptosis processes were not induced, despite that increase in extracellular glutamate concentration, enhanced lipid peroxidation, and inhibition in spatial memory were observed. However, this compound in female more strongly than in male rats affected the endocrine system and evoked a disturbance in the hematological parameters.
